# Heparan Sulfate Induces Necroptosis in Murine Cardiomyocytes: A Medical-*In silico* Approach Combining *In vitro* Experiments and Machine Learning

**DOI:** 10.3389/fimmu.2018.00393

**Published:** 2018-03-20

**Authors:** Elisabeth Zechendorf, Phillip Vaßen, Jieyi Zhang, Ahmed Hallawa, Antons Martincuks, Oliver Krenkel, Gerhard Müller-Newen, Tobias Schuerholz, Tim-Philipp Simon, Gernot Marx, Gerd Ascheid, Anke Schmeink, Guido Dartmann, Christoph Thiemermann, Lukas Martin

**Affiliations:** ^1^Department of Intensive Care and Intermediate Care, University Hospital RWTH Aachen, Aachen, Germany; ^2^Research Area Information Theory and Systematic Design of Communication Systems, RWTH Aachen University, Aachen, Germany; ^3^Chair for Integrated Signal Processing Systems, RWTH Aachen University, Aachen, Germany; ^4^Institute of Biochemistry and Molecular Biology, RWTH Aachen University, Aachen, Germany; ^5^Department of Medicine III, University Hospital RWTH Aachen, Aachen, Germany; ^6^Department of Anesthesia and Intensive Care, University Hospital Rostock, Rostock, Germany; ^7^Research Area Distributed Systems, Trier University of Applied Sciences, Trier, Germany; ^8^William Harvey Research Institute, Queen Mary University London, London, United Kingdom

**Keywords:** septic cardiomyopathy, necroptosis, apoptosis, Petri nets, modeling, optimization, small data

## Abstract

Life-threatening cardiomyopathy is a severe, but common, complication associated with severe trauma or sepsis. Several signaling pathways involved in apoptosis and necroptosis are linked to trauma- or sepsis-associated cardiomyopathy. However, the underling causative factors are still debatable. Heparan sulfate (HS) fragments belong to the class of danger/damage-associated molecular patterns liberated from endothelial-bound proteoglycans by heparanase during tissue injury associated with trauma or sepsis. We hypothesized that HS induces apoptosis or necroptosis in murine cardiomyocytes. By using a novel Medical-*In silico* approach that combines conventional cell culture experiments with machine learning algorithms, we aimed to reduce a significant part of the expensive and time-consuming cell culture experiments and data generation by using computational intelligence (refinement and replacement). Cardiomyocytes exposed to HS showed an activation of the intrinsic apoptosis signal pathway *via* cytochrome C and the activation of caspase 3 (both *p* < 0.001). Notably, the exposure of HS resulted in the induction of necroptosis by tumor necrosis factor α and receptor interaction protein 3 (*p* < 0.05; *p* < 0.01) and, hence, an increased level of necrotic cardiomyocytes. In conclusion, using this novel Medical-*In silico* approach, our data suggest (i) that HS induces necroptosis in cardiomyocytes by phosphorylation (activation) of receptor-interacting protein 3, (ii) that HS is a therapeutic target in trauma- or sepsis-associated cardiomyopathy, and (iii) indicate that this proof-of-concept is a first step toward simulating the extent of activated components in the pro-apoptotic pathway induced by HS with only a small data set gained from the *in vitro* experiments by using machine learning algorithms.

## Introduction

Severe injuries and systemic infection are the leading causes of death in intensive care units worldwide (1). Post-injury organ failure is defined as a life-threatening condition caused by a dysregulated host response to trauma or infection resulting in dysfunction and ultimately failure of many organs. The heart is one of the most frequently affected organs in the multiple organ failure syndrome associated with sepsis and trauma (2, 3). Several studies indicate that cardiac apoptosis, necrosis, or necroptosis play a pivotal pathophysiological role in cardiomyopathy associated with trauma or sepsis (4–7). Apoptosis describes a programmed, cysteinyl aspartate-specific protease (caspase)-dependent form of cell death, whereas necroptosis is a programmed caspase-independent form of necrosis. While the activation of caspase 3 leads to apoptosis induction, the activation of mixed lineage kinase domain-like (MLKL) and receptor-interacting protein (RIP) 3 results in necroptosis (8, 9). RIP3 and the pseudokinase MLKL form a necrosome to induce necroptosis. The underlying mechanisms and causative factors that induce cardiac apoptosis or necroptosis in trauma or sepsis, however, are largely unknown.

**Figure 1:**
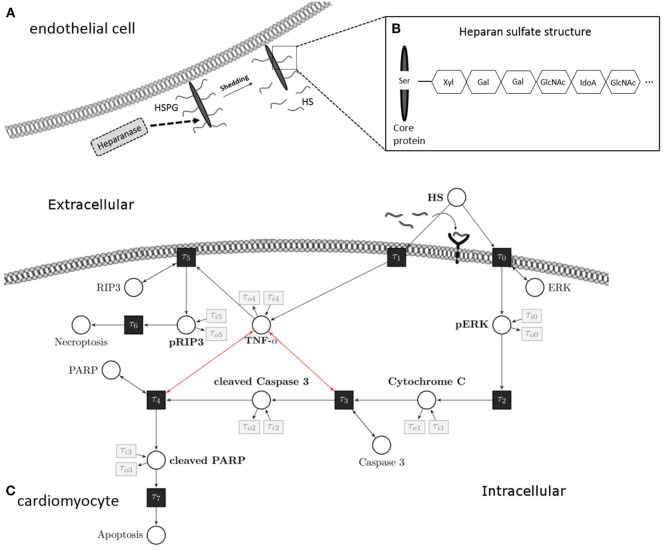
Heparan sulfate induces an apoptosis/necroptosis signal pathway. **(A)** HS fragments are cleaved by heparanase from a HS proteoglycan, which is localized on the plasma membrane of endothelial cells. **(B)** Structure of HS proteoglycan. **(C)** HS interacts with a pattern recognition receptor (i.e., toll-like receptor 4) localized on the cell surface of cardiomyocytes and activates a pro-apoptotic intrinsic pathway. The signaling cascade involves phosphorylation of ERK 1/2 resulting in the release of mitochondrial cytochrome C, which leads to cleavage and activation of caspase 3. In the next step of this pathway, PARP is cleaved and deactivated by activated caspase 3. Induction of TNF-α caused by HS inhibits the pro-apoptotic pathway and induces phosphorylation of RIP3 and necroptosis. These apoptosis and necroptosis signal pathways represent our Petri net model. White circles are places, and black rectangles are transitions. Unidirectional arcs indicate directed flows. Bidirectional arcs indicate read arcs, which influence transitions, but do not consume tokens. Modified from Martin et al., Sarrazin et al., and Maeda (13–15). HS, heparan sulfate; PARP, poly-(ADP-ribose) polymerase; ERK, extracellular signal-regulated kinase; RIP, receptor-interacting protein; TNF-α, tumor necrosis factor alpha; Ser, serine; Xyl, xylose; Gal, galactose; GlcNAc, N-acetylgalactosamine; IdoA, iduronic acid.

Tissue injury after trauma or infection results in an increased expression of pro-inflammatory cytokines, such as tumor necrosis factor alpha (TNF-α) and interleukin (IL)-6, both of which play a central role in the process of trauma and sepsis-associated cardiac dysfunction (10). Cytokines, such as IL-6, liberate and activate the endo-b-d-glucuronidase heparanase, a sheddase that specifically cleaves heparan sulfate (HS) fragments. HSs are highly sulfated glycosaminoglycans covalently attached to a core protein that is localized on the plasma membrane of endothelial cells (11). Once shed by heparanase, circulating HS fragments belong to the heterogeneous group of “danger/damage-associated molecular patterns (DAMPs)” (Figure 1). Indeed, patients with septic shock show elevated serum levels of HS fragments (12). Similar to molecules released by pathogens (pathogen-associated molecular patterns), HS interacts with pattern recognition receptors (e.g., toll-like receptors) on several cell types [including cardiomyocytes (12)], which results in an inflammatory response and mitochondrial dysfunction (11, 12). The role of HS in cardiac apoptosis and necroptosis, however, is unknown.

The use of *in vitro* experiments to investigate pathophysiological processes is complex, time-consuming, and expensive. A new and very promising solution to overcome these shortcomings of “classical” experimental techniques aimed at understanding biology is the modeling of system biological processes using computer-based methods (16–18). The access to data, however, is very limited in *in vitro* studies, facing a small data problem. Therefore, we established a methodology that involves expert knowledge in the modeling processes combined with the optimization of the unknown parameters using evolutionary algorithms (19–21). This family of algorithms is inspired from biological evolution and is adequate for optimization problems that lack full mathematical formalization between the tunable parameters and optimization objectives (22). Introduced by Carl Adam Petri in 1962, a Petri net is a simple graph that is built from places and transitions, which are interconnected by weighted arcs. In systems biology, places correspond to the measured amount of a substrate, transitions model changes in substrate [by ordinary differential equations (ODEs)], while weighted arcs model the influence of the specific transition on a place or *vice versa*. Petri nets are intuitive and offer both visualization and a mathematical formalism. Nevertheless, they are a very powerful tool for modeling complex (i.e., biological) processes and are, hence, emerging as promising and powerful tools in systems biology (23–26). As Petri nets have different abstraction levels, it is possible to model different biological processes (27). In this study, models were adapted and verified with available *in vitro* data sets. The structure of Petri nets can be extended to a system of ODE modeling the kinetic information. This allows us to extract a system of ODEs by defining transition functions between the substrates that are modeled by commonly used mass action kinetics (24, 28). Their application to biological processes, such as signal pathways, may have the potential to facilitate the generation of valuable data, which otherwise (with classical techniques) would be expensive and time-consuming to generate.

Thus, the aim of the present study was (i) to investigate if HS induces apoptosis or necroptosis in cardiomyocytes and (ii) to evaluate the signaling pathways involved. Additionally, we aimed to develop a proof-of-concept study for using Petri nets to simulate the missing data (different concentrations and time-points) by using evolutionary optimization to optimize the kinetic parameters and involving expert knowledge to model the structure of the network. Using this novel Medical-*In silico* approach, our data show (i) that HS induces necroptosis in cardiomyocytes by phosphorylation (activation) of RIP3 and (ii) indicate that this proof-of-concept is a first step toward simulating the extent of activated components in the pro-apoptotic pathway induced by HS with only a small data set gained from the *in vitro* experiments by using machine learning algorithms.

## Materials and Methods

### Cell Culture

As described previously (12, 29–31), HL-1 cells (murine cardiomyocytes) were cultured in 10 cm plates coated with a gelatin/fibronectin solution [5 mg/L fibronectin (Sigma, Munich, Germany), 0.02% (w/v) gelatin (Sigma)]. Cells were cultivated in Claycomb medium (Sigma) and incubated under an atmosphere of 5% CO_2_ and 95% air at 37°C. The medium was supplemented with 50 ml fetal calf serum (10%, Biochrom, Berlin, Germany), 5 ml norepinephrine (0.1 mM, Sigma), 5 ml l-glutamine (2 mM, Sigma-Aldrich, Munich, Germany) and 5 ml penicillin/streptomycin (Invitrogen, Carlsbad, CA, USA).

### Cell Stimulation

Cardiomyocytes were exposed to 10 μg/ml HS (Amsbio, Abingdon, UK) for 16 h. We used unstimulated cells as a negative control, and the cells exposed to 2.5 μM staurosporine as a positive control (AppliChem, Darmstadt, Germany).

### RNA Extraction and qPCR

RNA was isolated using the Trizol reagent, as described earlier (12, 30, 31). The following primers were used to analyze the relative mRNA expression of caspase 3 and TNF-α in the quantitative real-time PCR (StepOnePlus Real-Time PCR System; Thermo Fisher Scientific, MA, USA): caspase 3, 5′ CCAACCTCAGAGAGACATTC 3′ (for) and 5′ TTTCGGCTTTCCAGTCAGAC 3′ (rev) and TNF-α, 5′ TCCCCAAAGGGATGAGAAG 3′ (for) and 5′ GCACCACTAGTTGGTTGTC 3′ (rev). S7 was used as the reference gene: 5′ GGTGGTCGGAAAGCTATCA 3′ (for) and 5′ AAGTCCTCAAGGATGGCGT 3′ (rev). Relative quantification was performed by using Microsoft Excel (Microsoft, Washington, DC, USA).

### Western Blot Analysis

Cardiomyocytes were washed with PBS and lysed using Triton lysis buffer [300 mM NaCl (Roth, Karlsruhe, Germany), 20 mM TRIS (pH 7.4) (Merck, MA, USA), 1% Triton-X100 (Sigma-Aldrich), 200 mM PMSF (Roth), 1 mM DTT (Gerbu, Heidelberg, Germany), 2 mg/ml leupeptin (AppliChem), and 1 mg/ml pepstatin (AppliChem)] (12, 30). After 30 min on ice, the cells were centrifuged for 10 min at 4°C and 20,800 × *g*. The supernatant was transferred to a new tube, and the protein concentrations were determined by the Bradford method (Roti-Quant, Roth). Proteins were separated by 12% sodium dodecyl sulfate polyacrylamide gel electrophoresis. Gel electrophoresis was carried at 120 V. Separated proteins were transferred onto a polyvinylidene diflouride membrane. After blocking, membranes were incubated with specific primary antibodies against caspase 3 (Cell Signaling, Danvers, MA, USA), poly-(ADP-ribose) polymerase (PARP) (Cell Signaling), extracellular signal-regulated kinase (ERK) 1/2 (Cell Signaling), phospho-ERK 1/2 (Cell Signaling), cytochrome C (Cell Signaling), RIP3 (Bio-Rad), phospho-RIP3 (Ser232) (Abcam), MLKL (Cell Signaling), phospho-MLKL (Ser345) (Cell Signaling), and vinculin (Sigma). After incubation with a second antibody for 1 h at room temperature, proteins were detected with the ECL Prime Western Blotting Detection Reagent (GE Healthcare, Uppsala, Sweden) and the LAS-4000-System (Fujifilm, Tokyo, Japan).

### TdT-Mediated dUTP-Biotin Nick End Labeling (TUNEL)

This method was first described in 1992 by Gavrieli et al. to detect apoptotic cells (32). HL-1 cells were grown on glass coverslips coated with gelatin/fibronectin and exposed to HS or staurosporine for 16 h, respectively. After 16 h, the cells were washed three times with PBS and fixed with 500 μl of 4% PFA (Sigma-Aldrich) for 1 h at room temperature. Next, the cells were permeabilized (0.1% Triton X-100, 0.1% sodium citrate) for 2 min on ice. Cells were labeled with 5 μl of TUNEL-Enzyme and 45 μl of TUNEL-Label Solution (*In situ* Cell Detection Kit TMR red, Roche, Mannheim, Germany) for 1 h at 37°C in the dark. Cells only labeled with 45 μl of TUNEL-Label Solution were used as a negative control. After washing, the nuclei were stained with 15 μl of 4′,6-diamidino-2-phenylindole. LSM 710 confocal microscope (Zeiss, Oberkochen, Germany) was used for detection and further analysis.

### Fluorescence Flow Cytometry

Stimulated cardiomyocytes were washed three times with PBS on ice and harvested with a scraper. After centrifugation at 4°C and 500 × *g* for 5 min, the supernatant was discarded, and the cells were resuspended in binding buffer, stained with Annexin V (#550474) and 7-AAD (#559925), and then analyzed by Fortessa LSR (all BD Biosciences, NJ, USA). Unstained cells were used for gating the cells (upper left panel, Figure 4). The Annexin V and 7-AAD plots from the gated cells show the populations corresponding to viable cells (both Annexin V and 7-AAD negative, Gate I), early apoptotic cells (Annexin V positive and 7-AAD negative, Gate II), late apoptotic cells (both Annexin V and 7-AAD positive, Gate III), and necrotic cells (Annexin V negative and 7-AAD positive cells, Gate IV).

### Cell Vitality

Cells were grown on μ-slide 8-wells (IBIDI, Martinsried, Germany) to analyze cell viability. Cells stimulated for 16 h were washed with PBS and stained with propidium iodide (PI, BD Biosciences) and 5 μg/ml Hoechst (H3570, Invitrogen). Cell viability of cardiomyocytes was detected with live-cell imaging at 37°C and 5% CO_2_ in an incubator at an LSM 710 confocal microscope. ImageJ software was used to count the amount of dead cells.

### Statistical Analysis of the *In vitro* Measurements

The statistical analysis and the graphs of the relative protein expression were performed with GraphPad Prism 5 (GraphPad Inc., San Diego, CA, USA). An unpaired *t*-test or one-way ANOVA followed by the Bonferroni test was used for multiple comparisons with a significance level of *p* < 0.05. The data represent the mean ± SD for three independent experiments performed in triplicates.

### Medical-*In silico* Model

The measured *in vitro* data formed the basis for utilizing specific machine learning methods. The model was designed as a continuous Petri net (33). This allowed us to use the model for a computational prediction of involved signaling pathways describing the induced necroptosis in cardiomyocytes exposed to a given amount and time of HS. The structure of the Petri net was constructed analogous to the current understanding of the pathophysiological process of necroptosis. The mass action kinetics was implemented to model the pathophysiological process. This kinetics was represented by ODEs in the transitions of the Petri net (24). These ODEs were parameterized, where the parameters are adapted based on evolutionary optimization. As typical for results gained from *in vitro* measurements, our approach targeted the small data challenge. Thus, we optimized the parameters of the ODEs in order to minimize the mean square error of the given data samples to the simulated data.

The designed model is rather abstract and represents only a part of entire and complex pathophysiological process of apoptosis or necrosis. The model was constructed and optimized as follows (Figure 7):

- The pathophysiological process was comprehensively defined (Figure 7).
- Analog to the preceding description, the Petri net structure was derived.
- ODEs were extracted from the structure of the Petri net. The transition functions between the substrates were based on the mass action kinetics (Table 1).
  - The parameters of the kinetics (Figure 8) were optimized in a way that the simulated data fitted the measured data according to a minimum mean square error approach.
  - The output of the model was simulated based on the Matlab ODE solver (34) (Algorithm 1).
- Based on the system of ODEs, an optimization problem was formulated that incorporates the *in vitro* measurements.
- The ODEs were parameterized by Covariance Matrix Adaption—Evolutionary Strategy, an algorithm that minimizes the objective function (35, 36).
- A visualization of the time course was simulated by numerical integration from the parameterized ODEs.
- Finally, the model was analyzed and either accepted or previous design steps were refined.

**Table 1:** Ordinary differential equations (ODEs) of the proposed Petri net model.

Label	Place	ODE
Heparan sulfate	*p_0_*	$\dot{y}\left(p_{0},t\right)=\frac{\partial \text{HS}\left(t\right)}{\partial t}$
ph-ERK 1/2	*P_1_*	$\dot{y}(p_{1})=x_{11}x_{2}+x_{10}(x_{1}y{\left(p_{0}\right)}^{x_{9}})-x_{11}(x_{2}y{\left(p_{1}\right)}^{x_{11}})$
Cytochrome C	*P_2_*	y˙(p2)=(x13x3x26x25x26+x25+x26−x12x2)+x12(x2y(p1)x11)−x3y(p2)x13x13y(p2)y(p2)+x25(1+y(p8)/x26)
Cleaved caspase 3	*p_3_*	$\dot{y}(p_{3})=\frac{x_{15}x_{4}x_{28}}{x_{28}x_{27}+x_{28}+x_{27}}-\frac{x_{14}x_{3}x_{26}}{x_{26}x_{25}+x_{26}+x_{25}}+x_{3}y{\left(p_{2}\right)}^{x_{13}}\frac{x_{14}y(p_{2})}{y(p_{2})+x_{25}(1+y(p_{8})/x_{26})}-x_{4}y{\left(p_{3}\right)}^{x15}\frac{x_{15}y(p_{3})}{y(p_{3})+x_{27}(1+y(p_{8})/x_{28})}$
Cleaved PARP	*p_4_*	$\dot{y}(p_{4})=\left(x_{17}x_{5}-\frac{x_{16}x_{4}x_{28}}{x_{28}x_{27}+x_{28}+x_{27}}\right)+x_{16}x_{4}y{\left(p_{3}\right)}^{x_{15}}\frac{y(p_{3})}{y(p_{3})+x_{27}(1+y(p_{8})/x_{28})}-x_{17}(x_{5}y{\left(p_{4}\right)}^{x_{17}})$
ERK1/2	*p_5_*	$\dot{y}\left(p_{5}\right)=0$
Caspase 3	*p_6_*	$\dot{y}\left(p_{6}\right)=0$
PARP	*p_7_*	$\dot{y}\left(p_{7}\right)=0$
TNF-α	*p_8_*	$\dot{y}(p_{8})=x_{18}x_{17}+x_{20}\left(x_{6}y{\left(p_{0}\right)}^{x_{9}}\right)-x_{18}\left(x_{7}y{\left(p_{8}\right)}^{x_{18}}\right)$
pRIP3	*p_9_*	$\dot{y}(p_{9})=x_{21}x_{8}+x_{19}x_{7}+x_{19}\left(x_{7}y{\left(p_{8}\right)}^{x_{18}}\right)-x_{21}\left(x_{8}y{\left(p_{9}\right)}^{x_{21}}\right)$
RIP3	*p_10_*	$\dot{y}\left(p_{10}\right)=0$

*They are constructed according to the given definition in Figure 8 for general mass action kinetics. Two special inhibiting transitions are inspired by Michaelis–Menten inhibition (37). These are connected by red bidirectional arcs to TNF-α within the Petri net (Figure 1). *x_i_*’s are unknown, hence arbitrary selectable, parameters which will be used for optimization. Optimized parameters and constant terms *c*_1_ to *c*_8_, which are added to establish an equilibrium initial state, are given in the online supplementary. HS, heparan sulfate; PARP, poly-(ADP-ribose) polymerase; ERK, extracellular signal-regulated kinase; RIP, receptor-interacting protein; TNF-α, tumor necrosis factor alpha*.

**Algorithm 1:** Optimization Routine.

1: **procedure** Initialization()	
2: **y_0_** ←experimental start conditions	
3: **x_0_** ←initial guess	
4: **end procedure**	
5: **procedure** Simulation$\left(\dot{\text{y}},{\text{y}}_{0},\text{x},{\text{t}}_{0},{\text{t}}_{\text{end}}\right)$	
6: **y = y_0_**	
7: *t* = *t_0_*	
8: **while** *t* < *t_end_* **do**	
9: ${\text{y}}_{t}={\text{y}}_{\text{t}-1}+h\cdot \dot{\text{y}}$	➢ numerical integration with step size *h*
10: t = t + *h*	
11: **end while**	
12: **return** ${\text{y}}_{{\text{t}}_{\text{0}}:{\text{t}}_{\mathrm{end}}}$	
13: **end procedure**	
14: **procedure** Optimization(f_obj_(**x**))	
15: Initialization()	
16: **while** not terminated **do**	➢ terminate on satisfying solution ${\text{x}}^{*}$
17: **x** ←**x** + Δ**x**	➢ CMA-ES samples new candidates for **x**
18: Simulation($\dot{\text{y}},{\text{y}}_{0},\text{x},t_{0},t_{\mathrm{end}}$)	➢ Petri net simulation in time interval 0-24 h
19: r = f_obj_(**x**)	➢ evaluate objective function
20: CMA-ES ←(r,**x**)	➢ CMA-ES adjusts sampling of new **x**
21: **if** r is smallest r so far **then**	
22: x* ←**x**	
23: **end if**	
24: **end while**	
25: return x*	➢ best parameters to minimize the objective function
26: **end procedure**	

### Optimization Process

The optimization is mainly based on data acquired from *in vitro* experiments (39). Each protein and mRNA expression of the pathway is measured for a specific initial amount of induced HS after a specific time. For a mathematical formulation of the conducted optimization problem, i.e., fitting the model parameters to the experimental data, we defined a real valued observation function *g*(θ) that provides the experimental data to the algorithm. Since each place in the Petri net corresponds to a component of the signaling pathway, *g*(θ) is equivalent to a noisy measurement of place *p* within the model at time *t* and initial state *s* (40, 41). Each *in vitro* measurement is defined by the triple θ = (*p*,*t*,*s*), so that Θ is the set of all *in vitro* measurements. For instance, cleaved PARP is measured after 16 h within an *in vitro* experiment that was carried out for 10 μg/ml induced HS. Since cleaved PARP corresponds to place *p*_4_ in our model, the exemplary measurement is defined by θ = (*p* = *p*_4_, *t* = 16*h,s* = 10 μg/ml HS). To fit the model’s parameters, only a subset of all measurements Θ was used. This training set is denoted by Θ*_T_*. The remaining measurements are used for later validation of the acquired model. Following these definitions, the square errors between *in vitro* measurements and model simulation *y*(θ;***x***) (where ***x*** denote the parameter vector of the petri net model) are formulated by

$$f_{0}\left(\text{x}\right)={\underset{\theta \in \Theta_{T}}{\sum }|g\left(\theta \right)-y\left(\theta ;\text{x}\right)|}^{2}.$$

In order to satisfy the biological constraints, i.e., magnitudes of relative protein expressions, by the model, we added penalties to the objective function. Thereby, the model’s protein expressions are bound into reasonable magnitudes. To achieve this, the model should fulfill the upper bounds *u_p_* and lower bounds *l_p_* for simulated values of places *p*. The penalties are weighted by non-negative λ*_l_*, λ*_u_* and evaluated for all simulated times, places, and initial HS amounts, called Θ*_S_*, by

$$\begin{array}{ll} f_{u}\left(\text{x}\right) & =\lambda_{u}{\underset{\theta \in \Theta_{S}}{\sum }|{\left(y\left(\theta ;\text{x}\right)-u_{p}\right)}^{+}|}^{2}\\ f_{l}\left(\text{x}\right) & =\lambda_{l}{\underset{\theta \in \Theta_{S}}{\sum }|{\left(l_{p}-y\left(\theta ;\text{x}\right)\right)}^{+}|}^{2}. \end{array}$$

The penalties, which are added to the objective function, allow us to enforce the realistic modeling of the biological behavior. This leads to more realistic parameters. Furthermore, all parameters are restricted to positive real values (*x_lb_* > 0) with a limited magnitude (*x*_ub_ < ∞). All these assumptions result in the following optimization problem:

$$\begin{array}{l} \text{x}*=\underset{\text{x}}{\mathrm{arg min}}\;f_{\text{o}}\left(\text{x}\right)+f_{u}\left(\text{x}\right)+f_{1}\left(\text{x}\right)\\ \text{subject to }{\text{x}}_{\mathrm{lb}}<\text{x}<{\text{x}}_{\mathrm{ub}}. \end{array}$$

The above mentioned problem is solved with the CMA-ES optimizer (22, 35, 36). By this, the optimal set of parameters, x* that minimizes the objective function is utilized for numerical simulation of the model and time course prediction. The optimization routine is given in the listing of Algorithm 1. The source code for the presented procedure is made available on GitHub (https://github.com/Medical-In-silico/In-silico-Heparan-sulfate-induced-necroptosis-in-murine-cardiomyocytes, accessed February 4, 2018).

## Results

### HS Activates a Pro-Apoptotic Signal Cascade in Cardiomyocytes

**Figure 2:**
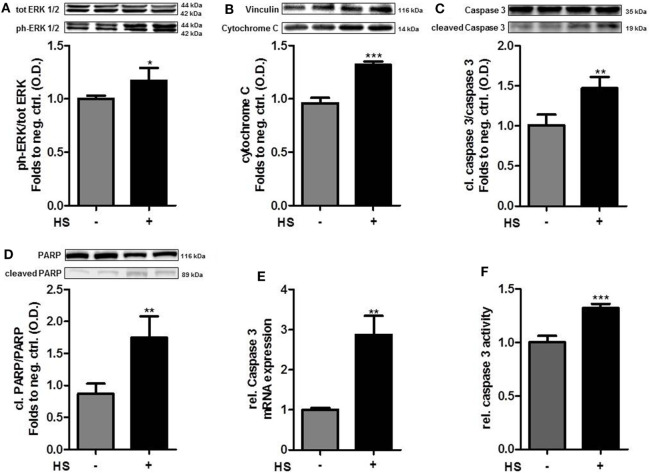
Heparan sulfate induces a pro-apoptotic pathway. HL-1 cells exposed to 10 μg/ml HS for 16 h showed a significant increase in protein expression of **(A)** phospho-ERK 1/2, **(B)** cytochrome C, **(C)** *cleaved* PARP, and **(D)** *cleaved* caspase 3, compared to unstimulated cells. Protein expression was normalized to unstimulated cells. **(E)** Relative mRNA expressions of HL-1 cells exposed to HS were analyzed by quantitative real-time PCR, compared to unstimulated cells. Caspase 3 mRNA expression was normalized to reference gene S7 and unstimulated cells. **(F)** Relative caspase 3 activity of cardiomyocytes exposed to HS, compared to unstimulated cells. The data represent the mean ± SD of triplicate samples for three independent experiments. HS, heparan sulfate; PARP, poly-(ADP-ribose) polymerase; p-ERK, phospho-extracellular signal-regulated kinase; statistical significance was performed by using unpaired *t*-test. *p < 0.05; **p < 0.01, and ***p < 0.001 vs. unstimulated cells.

The exposure of cardiomyocytes to HS for 16 h resulted in a significant increase in the phosphorylation of ERK 1/2 on Thr202/Tyr204 (*p* < 0.05; Figure 2A) and cytochrome C levels (*p* < 0.001; Figure 2B). HS stimulation also resulted in a significant activation (cleavage) of caspase 3 and inactivation of PARP compared to unstimulated cells (*p* < 0.01; *p* < 0.05; Figures 2C,D). Furthermore, expression of caspase 3 mRNA (*p* < 0.001; Figure 2E) and relative caspase 3 activity were significantly increased (*p* < 0.001; Figure 2F).

### HS Induces Necrosis, but Not Apoptosis in Cardiomyocytes

**Figure 3:**
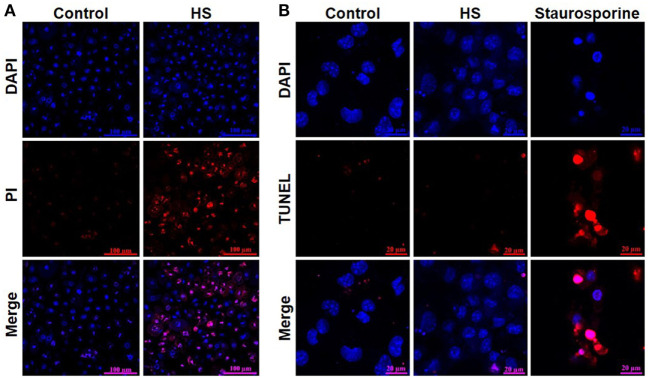
Heparan sulfate induces cell death but no apoptosis in cardiomyocytes. **(A)** HL-1 cardiomyocytes exposed to 10 μg/ml of HS for 16 h were stained with Hoechst and PI and compared to unstimulated cells (control) and analyzed with confocal live-cell imaging. Hoechst represent the cell nuclei (blue) and PI the death cells (red). **(B)** HL-1 cardiomyocytes exposed to HS for 16 h or 2.5 μM staurosporine were stained with DAPI and TUNEL and compared to unstimulated cells. DAPI represent the cell nuclei (blue) and TUNEL the apoptotic cells (red). HS, heparan sulfate; DAPI, 4′,6-diamidino-2-phenylindole; PI, propidium iodide; TUNEL, TdT-mediated dUTP-biotin nick end labeling.

**Figure 4:**
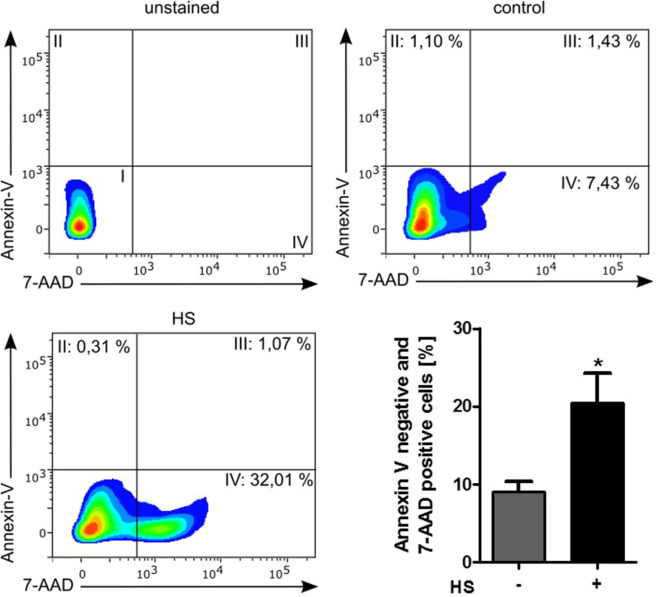
Cell death analysis by using FACS. HL-1 cells exposed to 10 μg/ml of HS and unstimulated cells (control) were stained with Annexin V and 7-AAD and then analyzed by fluorescence-based flow cytometry. Unstained cells were used for gating the cells. The Annexin V and 7-AAD plots from the gated cells show the populations corresponding to viable cells (both Annexin V and 7-AAD negative, Gate I), early apoptotic cells (Annexin V positive and 7-AAD negative, Gate II), late apoptotic cells (both Annexin V and 7-AAD positive, Gate III), and necrotic cells (Annexin V negative and 7-AAD positive cells, Gate IV). The bar chart represents the percentage (mean ± SD) of necrotic cells (Annexin V-negative and 7-AAD-positive cells) in unstimulated (control) and HS-treated HL-1 cells, respectively. Triplicate samples for three independent experiments are shown. HS, heparan sulfate; *p < 0.05 control vs. HS-treated HL-1 cells.

A total of 4,262 unstimulated cells and 4,243 HS-stimulated cardiomyocytes were examined with confocal live-cell imaging. The amount of dead cells was quantified by PI staining. Unstimulated cells showed a cell viability of 96.15%; however, cardiomyocytes exposed to HS showed a cell viability of 69.86% (Figure 3A). We next investigated the number of apoptotic cells. Notably, no apoptotic cells could be detected after exposure to HS, indicated by the lack of TUNEL-positive cells. In contrast, the stimulation with staurosporine for 16 h (as positive control) resulted in a significant amount of TUNEL-positive (apoptotic) cells (Figure 3B). Having shown that the exposure of HS resulted in a significant amount of cell death but no induction of apoptosis, we next investigated the amount of necrotic as well as apoptotic cells by using FACS. Unstained cells were used for gating the cells (upper left panel, Figure 4). The Annexin V and 7-AAD plots from the gated cells show the populations corresponding to viable cells (both Annexin V and 7-AAD negative, Gate I), early apoptotic cells (Annexin V positive and 7-AAD negative, Gate II), late apoptotic cells (both Annexin V and 7-AAD positive, Gate III), and necrotic cells (Annexin V negative and 7-AAD positive cells, Gate IV). Untreated cells (control) showed that the majority of cells were viable and non-apoptotic/necrotic (Gate I, upper right panel). In contrast to untreated cells (9.02 ± 1.34% Annexin V-negative and 7-AAD positive cells), the exposure of HL-1 cells to HS resulted in 20.40 ± 3.91% Annexin V-negative and 7-AAD-positive cells (Gate IV, upper right and lower left panel, *p* < 0.05).

### HS Induces Necroptosis, a Programmed form of Necrosis, in Cardiomyocytes

**Figure 5:**
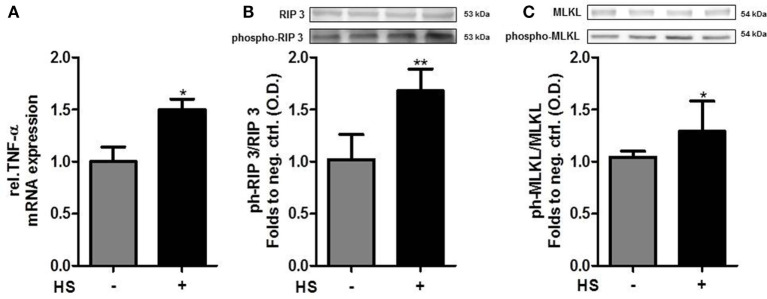
Heparan sulfate induces necroptosis in cardiomyocytes. **(A)** Relative caspase 3 mRNA expressions of HL-1 cells exposed to HS for 16 h were analyzed by quantitative real-time PCR, compared to unstimulated cells. Expression was normalized to reference gene S7 and normalized to unstimulated cells. **(B)** HL-1 cells exposed to 10 μg/ml HSs for 16 h showed a significant increase in phosphorylation of RIP3 and **(C)** MLKL compared to unstimulated cells. Protein expression was normalized to unstimulated cells. The data represent the mean ± SD of triplicate samples for three independent experiments. HS, heparan sulfate; RIP, receptor-interacting protein; MLKL, mixed lineage kinase domain-like; TNF-α, tumor necrosis factor alpha; *p < 0.05, **p < 0.01 vs. unstimulated cells.

Cardiomyocytes exposed to HS for 16 h showed a significant increase in the relative expression of TNF-α mRNA, compared to unstimulated cells (*p* < 0.05; Figure 5A). The exposure of cardiomyocytes to HS also resulted in a significant increase in the phosphorylation of RIP3 on Ser232 (*p* < 0.01; Figure 5B). Furthermore, cardiomyocytes exposed to HS showed a significant increase in the phosphorylation of MLKL on Ser345 (*p* < 0.05; Figure 5C) compared to unstimulated cells, indicating that activated MLKL and RIP3 form a necrosome.

### Time Course of PAPR Inactivation

**Figure 6:**
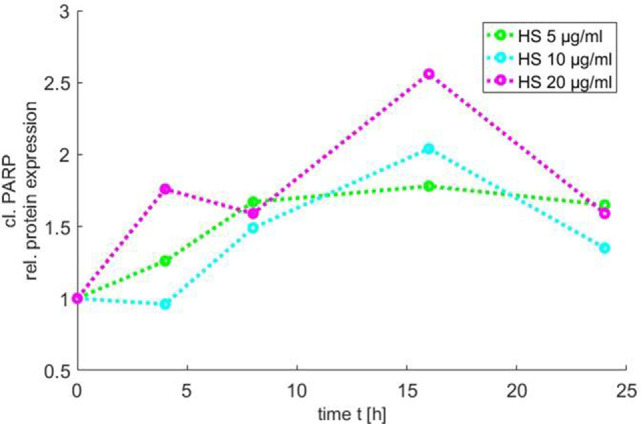
Time course of PARP inactivation. The relative PARP inactivation was analyzed by using Western blot and detected after 4, 8, 16, and 24 h after treatment of cardiomyocytes with 5, 10, or 20 μg/ml HS, respectively. HS, heparan sulfate; PARP, poly-(ADP-ribose) polymerase.

In order to use the new Medical-*In silico* approach, data from *in vitro* experiments of one component of the signal pathway induced by HS at different time points and concentrations were necessary. Thus, we generated *in vitro* a time course of the relative protein expression of cleaved PARP in cardiomyocytes exposed to 5, 10, and 20 μg/ml HS for 4, 8, 16, and 24 h, respectively, using Western blot analysis. The exposure of cardiomyocytes to either 5, 10, or 20 μg/ml HS resulted in an increase in PARP inactivation, with a peak of relative PARP inactivation after the exposure of cardiomyocytes to 20 μg/ml HS for 16 h (Figure 6).

**Figure 7:**
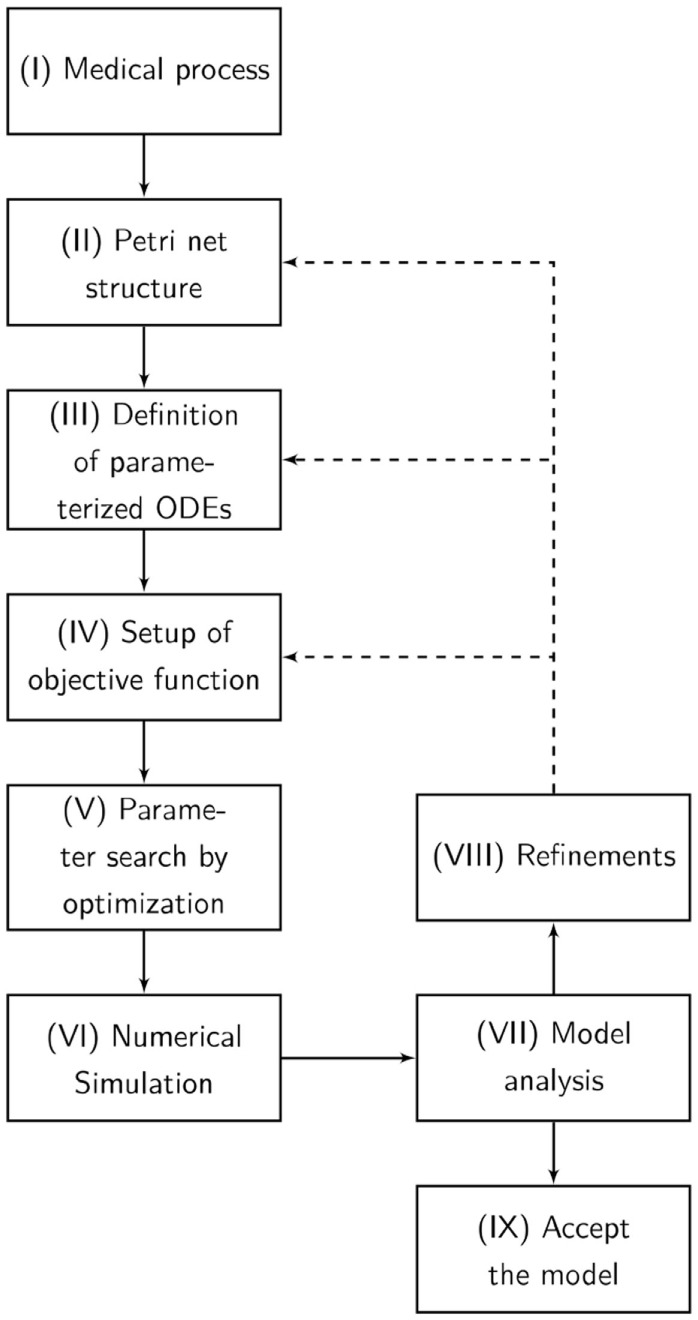
The flowchart presents our Medical-In silico approach. It shows all the undertaken processes from the pathophysiological process to the final model. (I) The pathophysiological process is comprehensively defined. (II) Analog to the preceding description, the Petri net structure is derived. (III) Ordinary differential equations (ODEs) are extracted from the structure of the Petri net. (IV) Based on the system of ODEs, an optimization problem is formulated incorporating in vitro measurements. We might enhance the objective function with some penalty functions that drive the model into biological boundaries. (V) The ODEs are parameterized by an evolutionary strategy algorithm that minimizes the objective function. (VI) A visualization of the time course is simulated by numerical integration from the parameterized ODEs. (VII) Finally, the model is analyzed and either accepted or previous design steps are refined.

**Figure 8:**
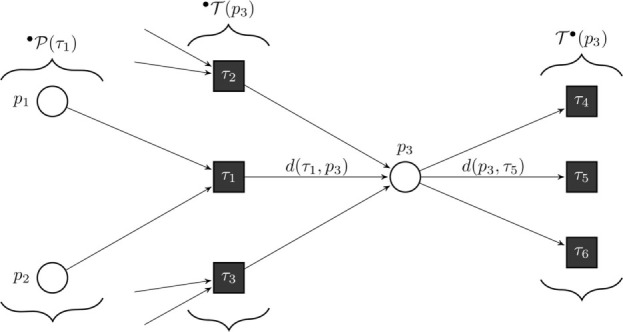
Formal definition of a continuous Petri net (CPN). Our model is a CPN. CPNs can be represented equivalently by ordinary differential equations and, thus, inherit dynamical behavior. The exemplary CPN shows our notation: °*T*(*p*) is the set of all incoming transitions of place *p*. *T*°(*p*) is the set of all outgoing transitions of place *p*. °*P*(τ) is the set of all places with an edge directed to transition τ. With this notation, we define the transition functions by $\frac{\partial y\left(p\right)}{\partial \text{t}}=\underset{\tau \in •T\left(p\right)}{\sum }d\left(\tau ,p\right)v\left(T\right)-\underset{\tau \in T•\left(p\right)}{\sum }d\left(p,T\right)v\left(T\right)$. In the case of general mass action kinetics (38), the transitions are given by $v\left(T\right)=x\left(T\right)\cdot \underset{p\in •P\left(T\right)}{\prod }\;y{\left(p\right)}^{d\left(\tau ,p\right)},$ where *x*(τ) are the kinetic rates.

### Simulated Time Course of Involved Components in Apoptosis and Necroptosis Pathway

**Figure 9:**
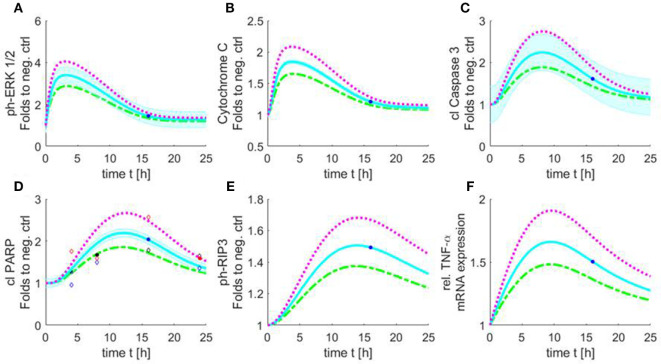
Simulated time course of all involved components of the apoptosis/necroptosis signal pathway induced by HS. Simulated time course of the components involved in intrinsic apoptosis and necroptosis signaling pathways for cardiomyocytes exposed to three different HS concentrations [5 (green), 10 (blue), and 20 (purple) μg/ml of HS]. The blue background represents the standard error of the measured data. The model was simulated based on relative cleaved PARP values. The filled points in graphs represent data used for training and the unfilled for validation. Simulated time course for relative protein expression of **(A)** phosphorylation of ERK 1/2, **(B)** cytochrome C, **(C)** cleaved caspase 3, **(D)** cleaved PARP, and **(E)** phosphorylation of RIP3 is shown. **(F)** Simulated data of relative TNF-α mRNA expression. HS, heparan sulfate; ph-ERK, phospho-extracellular signal-regulated kinase; PARP, poly-(ADP-ribose) polymerase; RIP, receptor-interacting protein; TNF-α, tumor necrosis factor alpha.

Next, we developed a proof-of-concept study for using Petri nets to simulate the missing data (different concentrations and time-points) by using evolutionary optimization to optimize the kinetic parameters and involving expert knowledge to model the structure of the network. Using the measured time course of cleaved PARP together with the results of the other measured components in cardiomyocytes exposed to 10 μg/ml HS for 16 h, we simulated the results for the missing time points and concentrations (Figure 9). Three dose–response curves over time were simulated [5 (green), 10 (blue), and 20 (purple) μg/ml of HS].

The simulation showed an increase in phosphorylation of ERK 1/2 in the first 2 h for all three HS concentrations. After 3 h, the phosphorylation of pERK 1/2 reached its maximum. The highest phosphorylation of pERK 1/2 could be detected after the exposure of 20 μg/ml of HS. After 3 h, a decrease in phosphorylation of pERK 1/2 was observed that reached baseline after 24 h (Figure 9A). The simulation also showed an increase in relative protein expression for cytochrome C with peak values being observed after 4 h (Figure 9B). Figure 9C shows the simulation of the activation of caspase 3 over time. The protein expression of the cleaved (activated) caspase 3 continuously increased until its peak at 8 h (independent of the investigated concentration) (Figure 9C). The simulated time course for cleaved PARP was (as expected) very similar to the measured cleaved PARP values (Figure 6), as more measured data were used for this parameter to train and validate the model. The simulated relative protein expression of cleaved PARP increased and peaked after 12 h. The simulation showed a slow decrease in the relative cleaved PARP protein expression after the peak values and did not reach the baseline within the simulated time (Figure 9D). The phosphorylation of RIP3 showed its maximum after 14 h with only a slight decrease for all three concentrations after reaching the peak values (Figure 9E). The simulation indicated an increase in the relative TNF-α mRNA expression during the first 8 h, which did not reach baseline values within the simulated time (Figure 9F).

## Discussion

Trauma and sepsis cause tissue injury, resulting in the release of DAMPs that are able to induce a pro-inflammatory signal cascade and the release of pro-inflammatory cytokines such as TNF-α (42). Several signaling pathways involved in apoptosis and necroptosis are linked to trauma- or sepsis-associated cardiomyopathy. However, the underling causative factors are still unknown. Using a novel Medical-*In silico* approach, this study shows (i) that HS induces necroptosis in cardiomyocytes by phosphorylation (activation) of RIP3, (ii) suggest that HS may have the potential as a therapeutic target in trauma- or sepsis-associated cardiomyopathy, and (iii) indicate that this proof-of-concept is a first step toward simulating the extent of activated components in the pro-apoptotic pathway induced by HS with only a small data set gained from the *in vitro* experiments by using machine learning algorithms.

### Activation of the Pro-Apoptotic Signaling Cascade by HS

Cell death caused by inflammation can be mediated by two processes: necrosis and apoptosis. It is well known that both, necrosis and apoptosis, lead to a loss of intact cardiomyocytes in cardiovascular diseases (10). Apoptosis represents a type of programmed cell death that is regulated by caspases cascade (8) and activated by two different pathways: the extrinsic and the intrinsic pathway (43). In the intrinsic pathway, the pro-apoptotic signaling cascade is proceeded by the release of mitochondrial cytochrome C. ERK 1/2 belong to the family of mitogen-activated protein kinases (MAPKs) which are involved in different cell processes and cell death (44). Phosphorylation and, thus, activation of ERK 1/2 by extracellular stimuli (such as DAMPs) results in the release of cytochrome C (45). Indeed, our data indicate that the exposure of HS to cardiomyocytes results in an increased expression of phosphorylation of ERK 1/2 and cytochrome C release (Figures 2A,B). Moreover, the exposure of HS to cardiomyocytes also resulted in the cleavage (activation) of caspase 3 (Figures 2C,E,F). The effector caspase 3 is a pro-apoptotic caspase that is activated by mitochondrial cytochrome C and induces apoptosis in its activated form. Furthermore, we found increased levels of the cleaved product of PARP, indicating the activation of the pro-apoptotic signal cascade (Figure 2D). Cleaved PARP is a by-product of the pro-apoptotic signal cascade. PARP, normally involved in DNA repair, is cleaved and subsequently deactivated by the pro-apoptotic-cleaved caspase 3 (46). Our results indicate that the induction of the pro-apoptotic cascade by HS may proceed through the intrinsic pathway (Figure 2) (47).

### HS Induces Necroptosis in Cardiomyocytes

Using TUNEL, we could not detect any apoptotic cardiomyocytes after the exposure to HS. Flow cytometry using Annexin V/7-AAD staining and determination of cell vitality using PI/Hoechst staining showed that HS (within 16 h) causes cell death secondary to necrosis, rather than apoptosis (Figures 3 and 4). Moreover, the simulation showed a peak of caspase 3 activation (Figure 9C) already after 8 h, suggesting that 16 h of HS exposure might be too long to detect apoptosis in cardiomyocytes, which indeed was not detectable in our *in vitro* experiments (Figures 3 and 4). Necrosis is known and described as an unprogrammed cell death; however, Degterev et al. described a programmed form of necrosis, named necroptosis (48). TNF-α is a pro-inflammatory cytokine that induces necroptosis by activation of RIP3 and MLKL (49, 50). More specifically, TNF-α induces the activation (phosphorylation) of RIP3, which forms a necrosome with MLKL and, hence, induces necroptosis (9). Indeed, the exposure of cardiomyocytes to HSs resulted in an increased, relative TNF-α mRNA expression (Figure 5A), confirming previous data of our group that showed higher TNF-α levels in the supernatant of cardiomyocytes exposed to HS (30). In line with this finding, we also found that RIP3 and MLKL activation was increased in cardiomyocytes exposed to HS for 16 h (Figures 5B,C). In addition, the simulation results showed the highest relative expression of TNF-α (Figure 9F) and the highest phosphorylation of RIP3 (Figure 9E) between 10 and 16 h, underlying the *in vitro* detected necroptosis after 16 h exposure to HS. The phosphorylation of RIP3 is essential and represents a pivotal pathway in the necroptosis induced by TNF-α, as phosphorylated RIP3 forms together with phosphorylated MLKL the necrosome, which ultimately induces necroptosis (51). Intracellular ATP levels also play a role in the crosstalk between apoptosis and necrosis, as lower ATP concentrations result in necrosis, while higher ATP concentrations drive apoptosis (52). Notably, we recently showed that the exposure of cardiomyocytes to HS results in lower ATP concentrations in cardiomyocytes (12), possibly secondary to excessive activation of PARP (53).

### Simulated Time Course

As the use of *in vitro* experiments to investigate pathophysiological processes is complex, time-consuming, and expensive, we aimed to investigate a new and very promising solution to overcome these shortcomings of “classical” experimental techniques using computer-based methods. Our Medical-*In silico* approach proposes an alternative to running a wide range of experiments with the objective of finding *interesting* points such as maximum and minimum, which is costly. The optimizer offers a function describing the relationship between the variables under investigation and time from a single training data set. Further experimental data points suggested by the *in silico* experiments can be used to further develop the model. This facilitates the accumulation of knowledge from experimental data and minimizes the number of needed experiments to be used by the optimizer. The simulated results (Figure 9) indicated that the optimization process offered solutions that fitted all the experimental data for all experiments. In addition, some of the experimental data sets were not used in the training process to be used for verification (Figure 9D). The mean deviation of these verification data was 20.49%. Given the low number of training and verification data relative to the system complexity, the verification process is non-conclusive; however, this verification process constitutes an indication to the system learning convergence, as a fully non-trained system has a higher mean SD (~50%).

### Limitation/Conclusion

As our investigation is limited to *in vitro* analyses, further *in vivo* studies have to confirm the role of HS-induced necroptosis in the pathophysiology of sepsis- and trauma-associated cardiomyopathy. Moreover, the simulated data were limited to a small set of data gained from *in vitro* experiments. In conclusion, our data showed for the first time that HS induces necroptosis in cardiomyocytes by phosphorylation (activation) of RIP3 and indicate that the used Medical-*In silico* approach (as a proof-of-concept) is a first step toward simulating the extent of activated components in the pro-apoptotic pathway with only a small data set gained from the *in vitro* experiments by using machine learning algorithms. Additionally, the simulation indicates that a Medical-*In silico* approach, as performed in this study, can help to identify the right time point for further measurements and indeed replaces further time-consuming and cost-intense *in vitro* experiments.

## Author Contributions

Conception and design: EZ, LM, GD, AS, and CT. *In vitro* experiments and data analyses: EZ, LM, TS, T-PS, AM, GM-N, OK, GM, and PV. Medical *in silico* experiments and data analyses: EZ, PV, JZ, GD, AS, LM, AH, and GA. EZ wrote the manuscript. Correction of the manuscript: EZ, PV, LM, CT, GM, GD, T-PS, and AS. All the authors reviewed and finally approved the manuscript.

## Conflict of Interest Statement

The authors declare that the research was conducted in the absence of any commercial or financial relationships that could be construed as a potential conflict of interest.
