# Corrigendum: Heparan Sulfate Induces Necroptosis in Murine Cardiomyocytes: A Medical-*in-Silico* Approach Combining *In Vitro* Experiments and Machine Learning

**DOI:** 10.3389/fimmu.2020.630463

**Published:** 2020-12-10

**Authors:** Elisabeth Zechendorf, Phillip Vaßen, Jieyi Zhang, Ahmed Hallawa, Antons Martincuks, Oliver Krenkel, Gerhard Müller-Newen, Tobias Schuerholz, Tim-Philipp Simon, Gernot Marx, Gerd Ascheid, Anke Schmeink, Guido Dartmann, Christoph Thiemermann, Lukas Martin

**Affiliations:** ^1^ Department of Intensive Care and Intermediate Care, University Hospital RWTH Aachen, Aachen, Germany; ^2^ Research Area Information Theory and Systematic Design of Communication Systems, RWTH Aachen University, Aachen, Germany; ^3^ Chair for Integrated Signal Processing Systems, RWTH Aachen University, Aachen, Germany; ^4^ Institute of Biochemistry and Molecular Biology, RWTH Aachen University, Aachen, Germany; ^5^ Department of Medicine III, University Hospital RWTH Aachen, Aachen, Germany; ^6^ Department of Anesthesia and Intensive Care, University Hospital Rostock, Rostock, Germany; ^7^ Research Area Distributed Systems, Trier University of Applied Sciences, Trier, Germany; ^8^ William Harvey Research Institute, Queen Mary University London, London, United Kingdom

**Keywords:** septic cardiomyopathy, necroptosis, apoptosis, Petri nets, modeling, optimization, small data

In the original article, there was an error in the **Author Contributions** section. The wording used to declare the contribution of Elisabeth Zechendorf was not clear.

The new **Author Contributions** section appears below.

Conception and design: EZ, LM, GD, AS, and CT. *In vitro* experiments and data analyses: EZ, LM, TS, T-PS, AM, GM-N, OK, GM, and PV. Medical *in silico* experiments and data analyses: EZ, PV, JZ, GD, AS, LM, AH, and GA. EZ wrote the manuscript. Correction of the manuscript: EZ, PV, LM, CT, GM, GD, T-PS, and AS. All the authors reviewed and finally approved the manuscript.

The authors apologize for this error and state that this does not change the scientific conclusions of the article in any way. The original article has been updated.

